# Reduction of Adolescent Idiopathic Scoliosis: A 13-Month Follow-Up

**DOI:** 10.7759/cureus.78669

**Published:** 2025-02-07

**Authors:** Justin M Dick, Sandy Spurgeon

**Affiliations:** 1 Physical Medicine and Rehabilitation, Clear Life Scoliosis Reduction and Chiropractic, Huntersville, USA; 2 Physical Therapy, Clear Life Scoliosis Reduction and Chiropractic, Huntersville, USA

**Keywords:** adolescent idiopathic scoliosis (ais), non-surgical orthopedics, physical medicine and rehabilitation, respiratory function tests, scoliosis reduction

## Abstract

Adolescent idiopathic scoliosis (AIS) is a complex spinal deformity that affects millions of people worldwide. Severe scoliosis is thought to be progressive. This case study examines the benefits of a comprehensive, non-surgical treatment protocol developed by the Chiropractic Leadership, Educational Advancement, and Research (CLEAR) Institute. The 14-year-old male had been diagnosed with a severe case of AIS (Risser 2). He presented with a Cobb angle of 42.4°. After 13 months of consistent adherence to the Clear Life Scoliosis Reduction treatment plan, the patient's Cobb angle was reduced to 23.8° and was accompanied by a marked improvement in the patient's physical function, including increased mobility, chest expansion, and enhanced respiratory capacity. The success of this case study can be attributed to a holistic approach to scoliosis management, which recognizes the importance of addressing the underlying biomechanical factors, musculature imbalances, and postural abnormalities that contribute to the condition. While surgical intervention can be the treatment of choice for severe cases of AIS, the findings of this case further support the growing body of research that highlights the efficacy of non-surgical interventions in the management of AIS, giving patients and healthcare providers an alternative to explore. Further studies are needed to explore the long-term efficacy of this approach and its potential to become a mainstream treatment option.

## Introduction

Adolescent idiopathic scoliosis (AIS) is a three-dimensional (3D) spinal deformity marked by abnormal curvature in the coronal plane and associated rotational misalignment of the spine [1]. This spinal deformity is found between 11 and 18 years of age [2]. This spinal deformity is measured by the Cobb angle. Scoliosis is classified as mild (Cobb angle: 10-24°), moderate (25-39°), or severe (≥40°) [3]. Mild-to-moderate scoliosis can lead to cosmetic deformities, back pain, functional limitations, and psychological challenges [4,5]. Severe scoliosis is associated with cardiac dysfunction and pulmonary complications [6-9].

Treatment of AIS includes chiropractic, physical therapy, braces, and surgical interventions. Bracing is recommended for AIS patients with a curvature of 25-45° [2]. Surgery is recommended for skeletally immature patients with a structural thoracic curve exceeding 45° or those experiencing continued curve progression [10].

The patient’s decision for care can be multifactorial. Nonsurgical treatments are less disruptive and allow patients to maintain normal activities with minimal risks. Nonsurgical treatments may not achieve significant cosmetic improvements, which could impact self-esteem. Nonsurgical options also pose minimal risks but may not prevent progression in more severe cases, potentially leading to long-term issues, such as chronic pain or respiratory problems.

Surgery involves significant recovery time. Surgical procedures carry inherent risks, such as infection, neurological injury, or complications from implants [11]. Surgery can provide more noticeable aesthetic correction, addressing concerns about appearance and potentially improving psychological well-being.

While surgical intervention, such as spinal fusion, has been the traditional treatment choice for severe cases of scoliosis, there is growing evidence that nonsurgical interventions can be effective in managing the condition and improving the overall quality of life for patients [12]. One such nonsurgical approach is the teachings of the Chiropractic Leadership, Educational Advancement, and Research (CLEAR) Institute through Parker University College of Chiropractic. This protocol combines abnormal spinal biomechanics, targeted exercises, postural re-education, and specialized bracing to address the underlying biomechanical imbalances that contribute to the progression of scoliosis [13]. The present report serves as an example illustrating the approach, an initial publication adding to nonsurgical approaches.

This case report focuses on a 14-year-old male patient with severe scoliosis and an initial Cobb angle of 42.4°. This patient presented with a Risser grade of 2, which demonstrates a high risk of progression [14]. Numerous outcome measures were taken during the initial consultation, reevaluated at completion of treatment, which included 20 in-office treatments, follow up at 40 days, and again at 13 months.

## Case presentation

Patient history and clinical findings

In April 2023, the family was informed that their 14-year-old son had scoliosis diagnosed by a local chiropractor. At this time, the patient was referred to a physiatrist. The physiatrist stated that “we need to get him in front of the scoliosis specialist" and referred him to a pediatric orthopedist. The orthopedist measured the scoliosis at 43°. The family was told that there was “not much that we can do.” A nighttime bracing was suggested until the scoliosis exceeds 50° and surgery can be performed. The family declined the night brace at this time.

Concurrently, the family pursued alternative options for their son. The case was co-managed in a private clinic offering alternative scoliosis management. The patient and family decided that they felt this “solution was the better solution." Thus, we decided not to get the brace through the insurance and purchased the ScoliBrace accompanied by 20 scoliosis visits. The patient's residence was located over an hour's drive away from the clinic and would need weekly co-management by the local chiropractor. The patient was encouraged to continue orthopedic appointments.

The patient presented with a family history of scoliosis, that being a younger female sibling. The patient had daily neck and back pain with fatigue that increased as the day progressed. The patient denied radiating pain to the upper or lower extremities. This pain was reduced with rest. This did not limit any activities of daily living. The patient denied any other chronic conditions.

The patient's blood pressure measured 132/77 mmHg with a heart rate of 69 beats per minute. A height was measured at 156 cm. Visual inspection of the patient revealed a right head tilt with right rotation and translation, along with a right high hip and a left high shoulder. The cervical and thoracolumbar range of motion was hypomobile in flexion, extension, right and left rotation, and right and left lateral flexion. Palpation revealed areas of muscle spasm paraspinal throughout the cervical spine and extended into bilateral levator scapulae. An increase in muscle tone was seen in the right quadratus lumborum. Supine leg check measured a left short leg of 1/4 inch. Table 1 presents the physical and orthopedic examination results.

**Table 1:** Physical and orthopedic examination results of the pre-treatment assessment WNL: Within normal limits

Characteristics	Pre-treatment
Forward head posture (measured)	1.0 inch
Scoliometry evaluation	
Dorsal, flexion	4 degrees on the right
Dorsal lumbar, flexion	10 degrees on the right
Lumbar, flexion	5 degrees on the right
Dorsal, prone	5 degrees on the left
Dorsal lumbar, prone	5 degrees on the right
Lumbar, prone	10 degrees on the right
Timed 1-legged test	
Left (eyes open)	WNL (30 seconds, minimal sway)
Right (eyes open)	WNL (30 seconds, minimal sway)
Timed 1-legged test	
Left (eyes closed)	7 seconds, fall
Right (eyes closed)	6 seconds, fall
Thoracic lordotization	T1-T12
Cervical flexion test (measured)	Chin to chest 2.0 inches
Modified scoliosis cox test (prone)	
Left	Positive at 45 degrees
Right	Positive at 45 degrees
Spirometry	2100 cc’s
Chest expansion (measured)	2.0 inches
Functional rating index	10

Orthopedic examinations revealed negative superficial abdominal reflex, shoulder depression, foraminal compression, cervical distraction, and straight leg raiser.

The asymmetry of the hips, shoulders, and spinal muscles and scoliometer readings are strong indicators of scoliosis. The abnormal combined findings above confirmed the diagnosis of scoliosis.

Radiographic findings

Thorough radiographic assessment was conducted by a licensed chiropractor adhering to all federal and state guidelines to evaluate the severity and extent of the patient's spinal deformity. Anterior-posterior and lateral X-rays were taken of the cervical, thoracic, and lumbar spines. There were no pathologies found that warranted referral for additional testing. The X-rays were evaluated using the PostureRay® (PostureCo®, Inc., Trinity, FL) computerized digitization tool.

**Figure 1:**
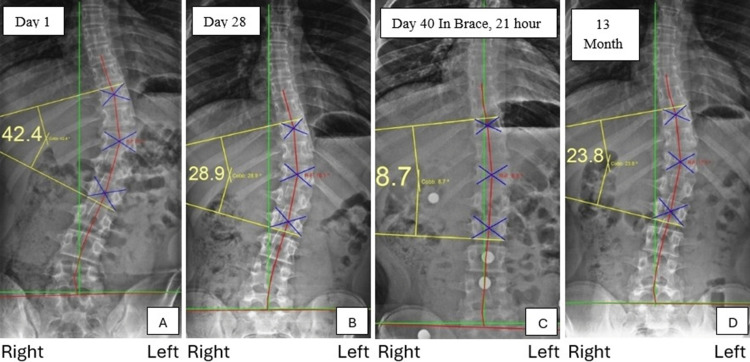
AP scoliosis radiographs Anterior-posterior (AP) full spine radiographs. Panel A is before treatment. Panel B is after treatment. Panel C is the 40-day brace radiograph. Panel D is the 13-month follow-up. The yellow lines are the Cobb measurement of scoliosis. The green lines are the ideal balance of the ideal spine. The red lines are the Risser-Ferguson analysis of the patient’s spine. The results show improvements in the Cobb angle and improved spinal coronal balance. All measurements were examined with PostureRay® software.

All radiographs were digitally measured using PostureRay® software, which uses machine-learning-assisted radiographic parameter mensuration (Figure 1). The patient’s initial Cobb angle was 42.4°, indicating a severe case of adolescent idiopathic scoliosis with a Risser grade of 2 [15]. This was reduced to 28.9° after treatment and reduced further to 23.8° at the 13-month follow-up.

These radiographic findings, in combination with the patient's clinical presentation, reaffirmed the diagnosis of severe adolescent idiopathic scoliosis and the need for a comprehensive, nonsurgical intervention to address the underlying biomechanical imbalances. These demonstrate a high risk of progression as the patient is a Risser 2 and diagnosed with severe scoliosis.

Treatment protocols and frequency

After the initial evaluation, the patient was enrolled in a comprehensive treatment program for 20 in-office treatments over five weeks with a ScoliBrace® (ScoliBrace, Kogarah, NSW, Australia). The patient performed each treatment protocol consecutively at each visit, which followed the “mix, fix, and set” of the CLEAR Institute [13]. Each treatment session lasted approximately 120 minutes. "Mix" is designed to “warm” the body up with spinal mobility exercises. "Fix" is designed to mechanically move the spine with the objective of realigning the spine. "Set" is designed to rehabilitate the spine and maintain the new alignment. This process is utilized in each treatment session.

**Figure 2:**
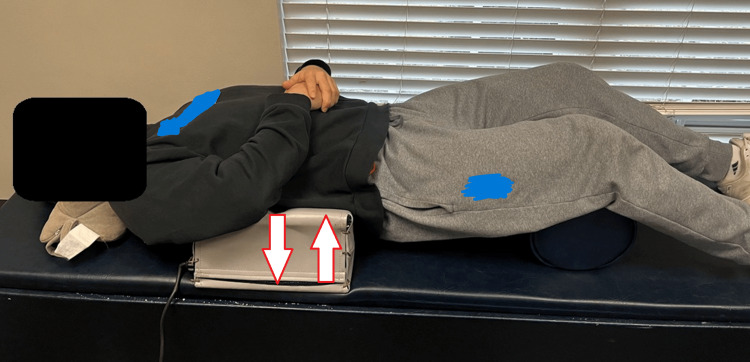
Mechanical drop piece (mechanical traction) When placed perpendicular to the spine, it will cause a rotation of the spinous processes and reduce the rib hump. It also is utilized in the thoracolumbar and sacral-coccygeal areas to assist in the correction of the alignment. This was performed for 20 minutes each session (model used for demonstration).

**Figure 3:**
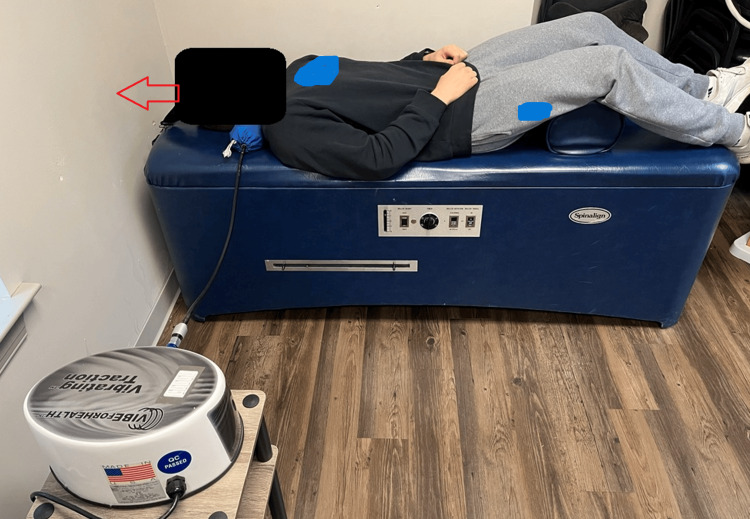
Cervical vibrating traction (mechanical traction) Cervical vibrating traction (mechanical traction) to improve cervical lordosis. Mechanical traction forces are used to create a degree of tension of soft tissue, specifically ligaments and disc and to allow for separation between joint surfaces. A 5-pound Y-axis traction was used. The vibration at 4.5 Hz allows for the relaxation of the ligaments and disc, so they can adapt to the new alignment with spinal support. This was performed for 20 minutes each session (model used for demonstration).

**Figure 4:**
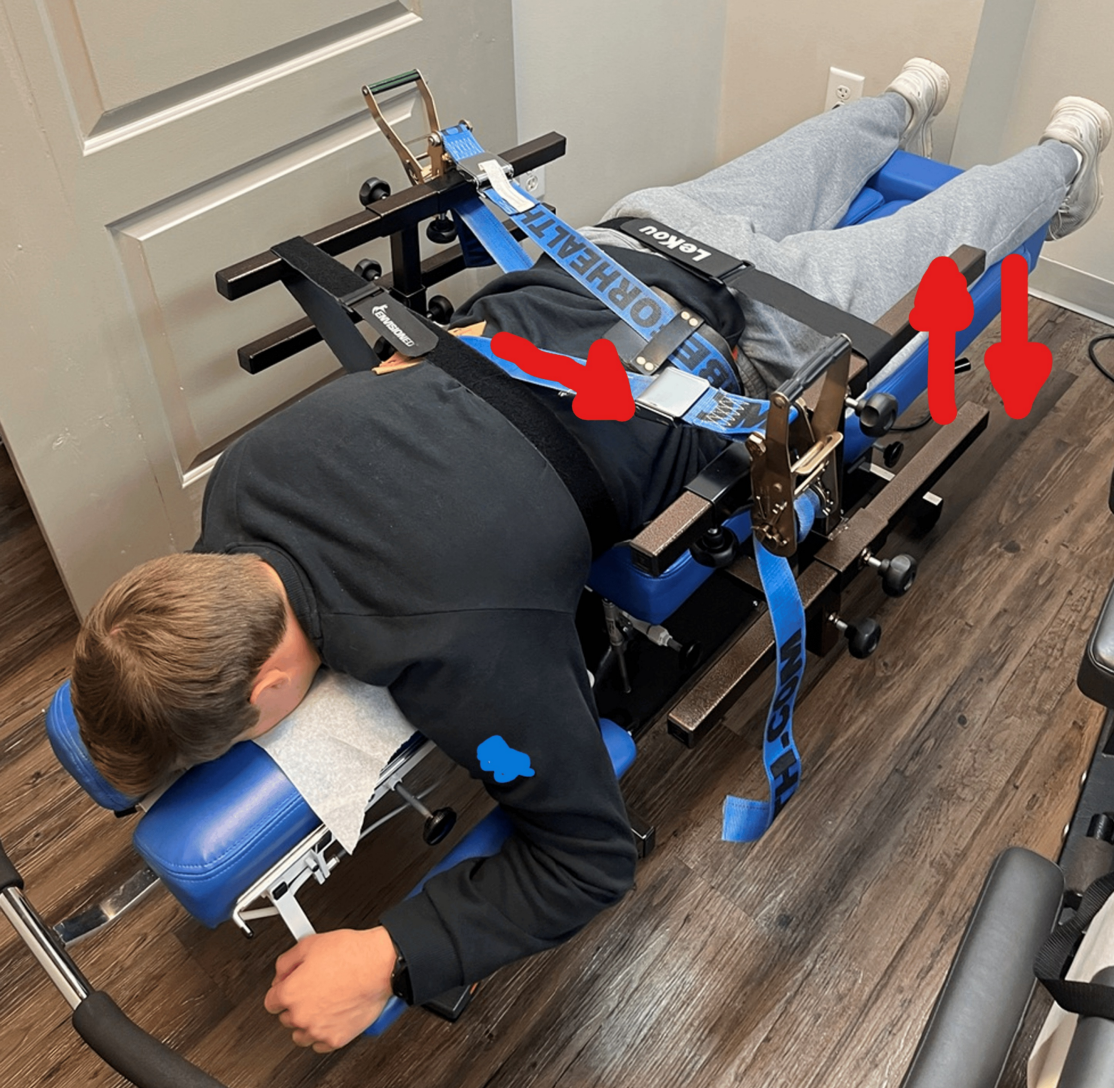
Flexion/distraction table Eckard table (mechanical traction). This is done with mirror image positioning of the patient based on their X-ray configuration. The Eckard table will provide motion and mechanical traction. This therapy will create a range of motion and flexibility, and mechanical traction will be a force used to allow for the separation between joint surfaces. This was performed for 20 minutes each session (model used for demonstration).

The following activities were performed under the direct supervision of a physician. The “Mix” phase included the following. Active rehabilitation chair warm-up exercises were utilized to increase disc metabolism and flexibility through motion. Cervical lordotic traction was performed each visit to improve the cervical spine, decrease the forward head posture, and aid with disc metabolism with motion. Core muscle stimulation was performed to reduce muscle guarding and decrease muscle toxins. The mechanical drop piece (Figure 2) was used to apply an impulse force of 6 Hz in neuromuscular education in regard to movement, balance, coordination, and kinesthetic sense posture and/or proprioception. Y-axis mechanical traction was integrated by applying a controlled pulling force to the spine, stretching and elongating the surrounding soft tissues. This stretching effect helps relieve tension and tightness in the muscles, ligaments, and fascia, promoting improved spinal alignment [16]. This concept was also used in the cervical vibrating traction (Figure 3). These and the Eckard table (Figure 4) were utilized on each visit.

The patient then received the “Fix” adjustments. Cervical adjustments were exclusively given using an ArthroStim® (IMPAC Inc., Salem, OR). At each visit, supine leg checks were utilized to determine atlas alignment. Adjustments were only performed on the atlas until the legs were balanced in the supine position [17]. Each adjustment also included the following: manual anterior T1-T12 and sacrum adjustments, tapotement paraspinal muscles (12 Hz), cervical spine in flexion, and extension adjustments (according to physical and radiographs).

**Figure 5:**
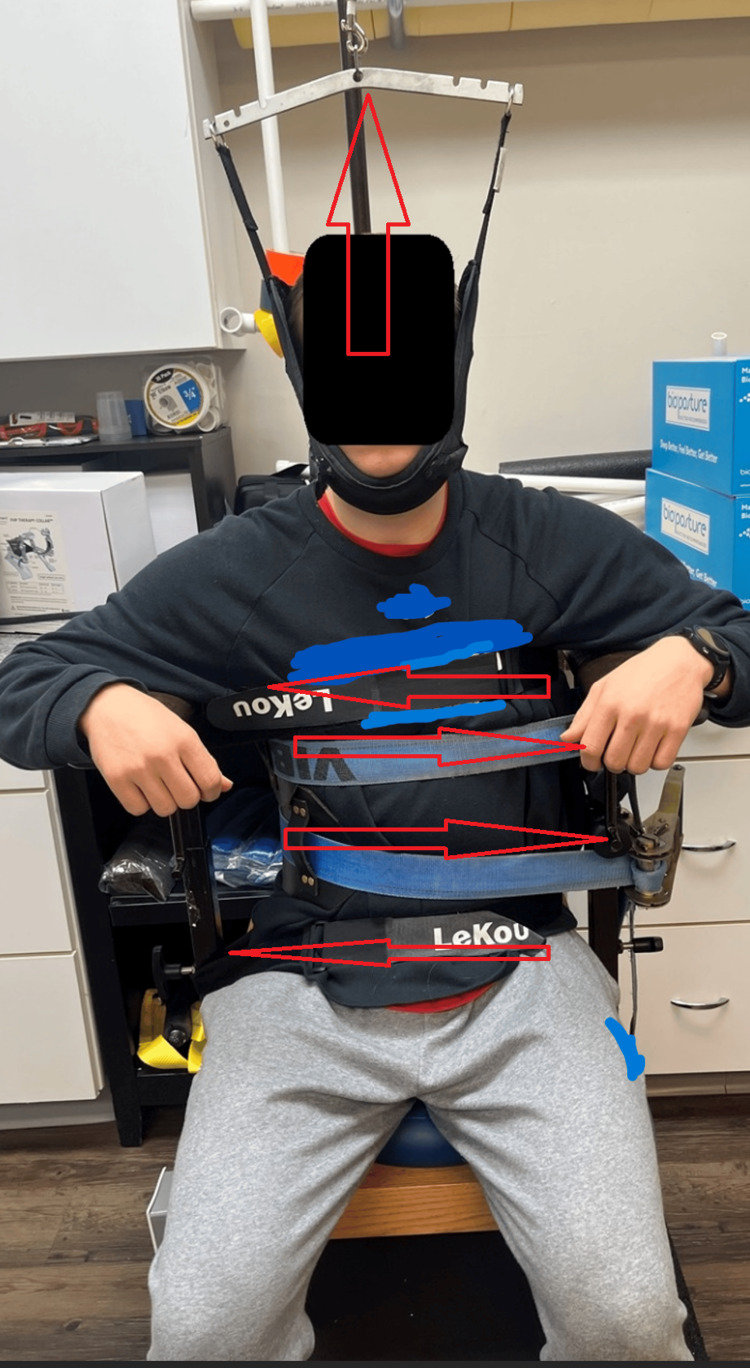
Scoliosis traction chair Scoliosis traction chair (mechanical traction) (whole-body vibration) (proprioceptive neuromuscular re-education) is a combination of whole-body vibration with mechanical traction with opposing forces for Cobb angle reduction. The patient is seated in the scoliosis chair with 10 pounds of Y-axis traction, lateral pulling straps to reduce the Cobb angles, and whole-body vibration with simultaneous vertical traction. Placement in the scoliosis chair is verified by X-rays. The purpose is meant for movement, balance, coordination, kinesthetic sense, posture for sitting, or standing activities. This was performed for 30 minutes each session (model used for demonstration).

**Figure 6:**
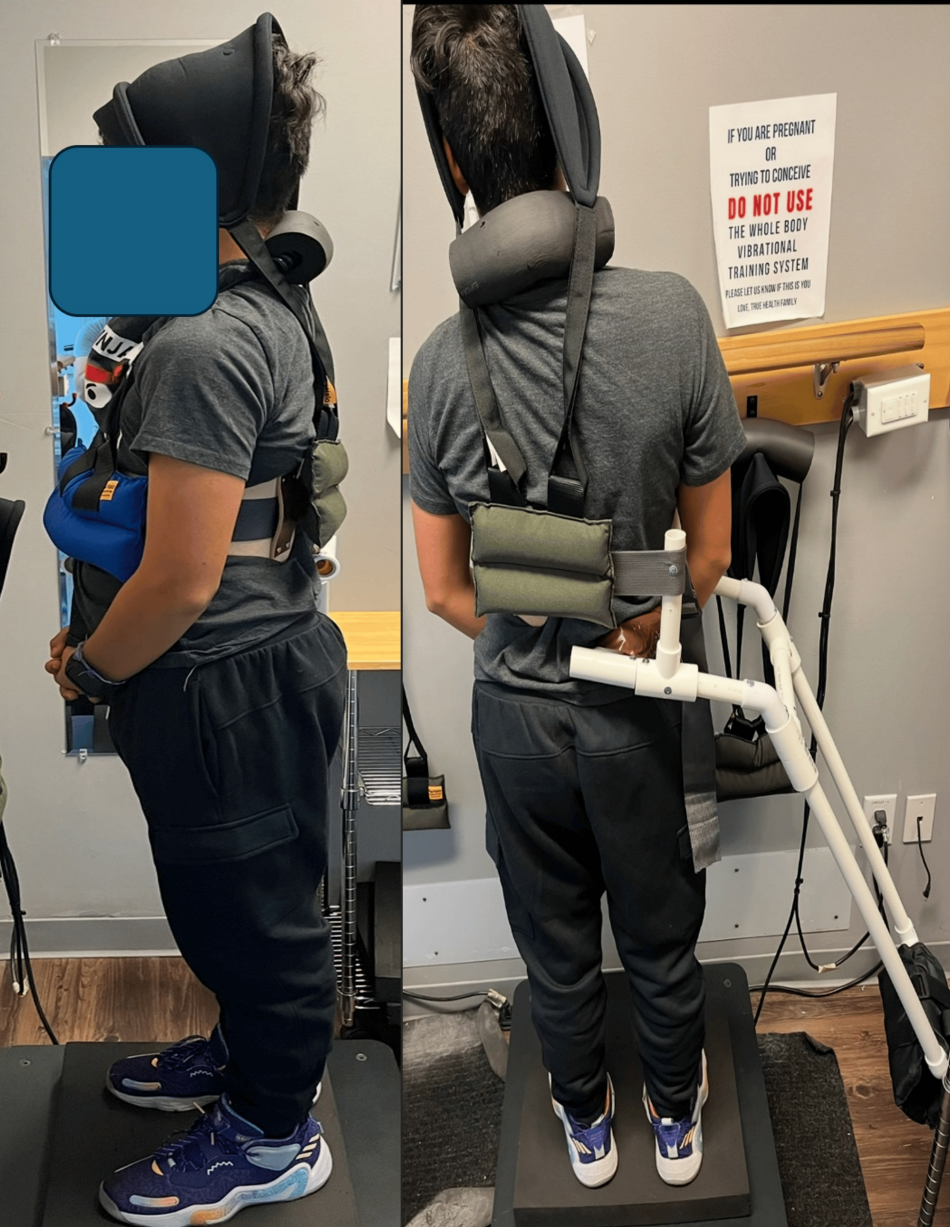
Spinal weighting (proprioceptive neuromuscular re-education) Spinal weighting (proprioceptive neuromuscular re-education-PVC cantilever, hood and chest weight). The patient wore uneven weights to reduce spinal misalignments while performing balance ans proprioceptive exercises, which are a type of vibration training on a foam pad (which is an uneven base), as well as on a vibrating platform. The purpose is meant for movement, balance, coordination, kinesthetic sense, and posture for sitting or standing activities. Evaluation and training is provided to a patient whose balance has been impaired by neurological, muscular, or skeletal abnormality and is reasonably expected to improve the patient's ability to balance. This was performed for 10 minutes each session and daily for home use.

The “Set” phase utilized the scoliosis traction chair (Figure 5) and whole-body vibration (Figure 6).

The patient performed specific spinal isometric exercises and scoliosis stretching exercises to develop strength, endurance, range of motion, and flexibility. These exercises are patient-specific and based on their X-rays with instructions. These included exercise cushion (lateral lean), spinal twist, cervical over-the-door traction, cervical extension, standing spinal twist, chest expander, and cervical flexion exercise. Two sets of 20 repetitions of each were performed every office visit and as daily home care.

During the sixth in-office treatment, the patient was given the following equipment for home therapy: PVC cantilever with 10-pound weight, 5-pound hood weight, and 8-pound chest weight. The cantilever was reduced to 6 pounds during the 8th in-office visit with improved patient tolerance. The cantilever was made specifically for this patient as a fulcrum to induce the righting reflex, improve posture, and restore spinal alignment. During the 90-day follow-up in-office visit, the cantilever was increased to 10 pounds with good tolerance for 10 minutes each session.

In-office acquisition of required information, fitting, and instructions in the proper use of a ScoliBrace® for the management of their scoliosis during the 13th in-office visit: ScoliBrace® is designed to prevent the patient's scoliosis from progressing and, in some cases, may help reduce the magnitude of spinal curvatures. The patient was instructed to wear the brace for two hours the first day, to increase by two hours each day until 21 hours were achieved, and to continue wearing the brace daily until skeletal maturity was achieved.

Once the patient completed in-office care, he returned home and continued one adjustment each week, co-managed by a chiropractor. The patient continued to be seen by their orthopedist.

Results

The patient underwent 20 in-office treatments over five weeks, which included exercise therapy, manual and mechanical traction, proprioceptive neuromuscular re-education, and manual and ArthroStim® adjustments. Initial subjective and objective outcome measures were reassessed for post-treatment and 13-month follow-up by the same chiropractor who conducted the initial evaluation. Please see Table 2 for the comparative findings.

**Table 2:** Physical and orthopedic examination results after treatment and 13-month follow-up ROM: Range of motion; WNL: Within normal limits

Items	End of Treatment (visit 19 of 20)	13-month follow-up
Height	158.5 cm	160 cm
Head tilt	Right	Negative
Head rotation	Right	Right
Head lateral translation	Right	Negative
High shoulder	Right	Negative
High hip	Right	Right
Forward head posture (measured)	0.75 inch	0.5 inch
Scoliometry evaluation		
Dorsal, flexion	1 degree on the right	1 degree on the right
Dorsal lumbar, flexion	6 degrees on the right	0
Lumbar, flexion	5 degrees on the right	2 degrees on the left
Dorsal, prone	0	1 degree on the right
Dorsal lumbar, prone	0	0
Lumbar, prone	9 degrees on the right	5 degrees on the right
Timed 1-legged test		
Left (eyes open)	WNL (30 seconds, minimal sway)	WNL (30 seconds, minimal sway)
Right (eyes open)	WNL (30 seconds, minimal sway)	WNL (30 seconds, minimal sway)
Timed 1-legged test		
Left (eyes closed)	6 seconds, fall	5 seconds, fall
Right (eyes closed)	15 seconds, fall	9 seconds, fall
Cervical ROM		
Flexion	WNL (55 degrees)	WNL (55 degrees)
Extension	WNL (65 degrees)	WNL (65 degrees)
Right rotation	Hypomobile	Hypomobile
Left rotation	Hypomobile	Hypomobile
Right lateral flexion	Hypomobile	Hypomobile
Left lateral flexion	Hypomobile	Hypomobile
Thoracolumbar ROM		
Flexion	Hypomobile	Hypomobile
Extension	Hypomobile	Hypomobile
Right rotation	Hypomobile	Hypomobile
Left rotation	Hypomobile	Hypomobile
Right lateral flexion	Hypomobile	Hypomobile
Left lateral flexion	Hypomobile	Hypomobile
Thoracic lordotization	T2-T10	T2-T10
Cervical flexion test (measured)	Chin to chest 0.0 inches	Chin to chest 0.0 inches
Modified scoliosis cox test (prone)		
Left	Positive at 60 degrees	Positive at 60 degrees
Right	Positive at 60 degrees	Positive at 60 degrees
Spirometry	2600 cc’s	3700 cc’s
Chest expansion (measured)	2.13 inches	3.0 inches
Functional rating index	12	9

The patient's daily recommendations were followed 70% of the time. Brace wear was recorded 15-20 hours a day.

Post-treatment radiographic outcome findings: Following the in-office treatments, the patient abstained from further treatment or exercises for at least 12 hours prior to reassessment. This break in care was implemented to eliminate any potential influence of residual effects from the previous treatments on the radiographic results. The post-treatment radiographs were obtained by the same physician who performed the initial imaging, adhering to identical patient instructions to ensure neutral and natural positioning. This consistency minimized the risk of external interference or examiner bias in patient positioning during the procedure (Figure 1).

## Discussion

The outcomes observed in this case study highlight the potential of nonsurgical interventions in the management of severe adolescent idiopathic scoliosis [18]. Severe scoliosis is thought to be progressive [18], but the combined efforts of restoring biomechanical alignment, specialized bracing, targeted exercises, and postural re-education have been shown to effectively reduce the Cobb angle and improve the overall quality of life for this patient [19-21]. Of additional significance is the fact that this patient has likely not achieved skeletal maturity, and previous research has shown that with skeletal growth typically comes an increase in the severity of scoliosis [4].

The field of nonsurgical interventions has become increasingly prevalent in the medical community, with various healthcare professionals, including medical doctors, physical therapists, and chiropractors seeking to provide alternative treatments to their patients. However, the widespread adoption of these interventions for complex spinal deformities, such as scoliosis, has been hindered by several key limitations. These include a lack of training, education, and understanding among healthcare providers.

Medical, physical therapy, and chiropractic programs can benefit from additional training and resources to enhance students' understanding of nonsurgical interventions, including manual therapy, exercise therapy, and other conservative treatments [22]. Consequently, healthcare providers may lack the necessary knowledge and skills to effectively implement these interventions, which can result in suboptimal outcomes for their patients [23].

Nonsurgical scoliosis treatment is often managed through physical therapy and chiropractic care. These treatments aim to improve spinal dynamics, reduce pain, and enhance the patient’s posture. The nonsurgical treatments have notable limitations in effectively addressing scoliosis.

One limitation is the absence of standardized, evidence-based treatment protocols tailored for scoliosis. Physical therapy often emphasizes bilateral strengthening and core stabilization. These do not adequately address the asymmetrical and 3D nature of scoliosis [24]. Many physical therapists receive general musculoskeletal training but do not specialize in scoliosis-specific rehabilitation techniques [25,26].

Similarly, general chiropractic manipulative therapies may be ineffective in restoring proper biomechanics of the spine [26,27]. Standard chiropractic education is primarily centered on vertebral subluxation theories and segmental alignment, rather than the larger biomechanical factors at play in scoliosis [26]. Without advanced scoliosis-specific training, many chiropractors apply generic spinal adjustments that are not biomechanically appropriate for scoliosis patients [28]. This further emphasizes the need for expanded scoliosis education within chiropractic programs to ensure effective treatment strategies.

Addressing these gaps requires a collaborative effort among healthcare professionals to integrate specialized scoliosis training into medicine, physical therapy, and chiropractic education. By incorporating evidence-based, scoliosis-specific protocols, practitioners can provide more targeted and effective treatment, improving patient outcomes. Furthermore, interdisciplinary approaches that combine expertise from multiple fields can lead to more comprehensive care strategies. Ultimately, enhancing scoliosis management through specialized education and collaboration will help patients achieve a better quality of life [25,29,30].

The results of this case report are consistent with the findings of previous research on the efficacy of scoliosis-specific exercises and chiropractic interventions in the management of adolescent idiopathic scoliosis [12,31]. These studies have demonstrated that nonsurgical approaches can lead to significant improvements in spinal alignment, respiratory function, and physical capacity.

This case is limited by its focus on a single patient, which prevents the ability to draw generalized conclusions from the results. Therefore, these results cannot be universally expected in all cases of adolescent idiopathic scoliosis. Although a 13-month follow-up was reported, the long-term sustainability of spinal corrections remains uncertain. Based on the above results, it is reasonable to infer that ongoing maintenance treatments will be required at a minimum until skeletal maturity is reached. In-office treatment time and cost restraints may also contribute to a limiting factor.

## Conclusions

The reduction of thoracolumbar scoliosis and improved coronal alignment can be achieved utilizing CLEAR Institute protocols. This case offers additional evidence supporting the potential effectiveness of conservative treatments for abnormal spinal conditions.
